# Genomic insights into indole-3-acetic acid catabolism in the marine algae-associated bacterium, Marinomonas sp. NFXS50

**DOI:** 10.1099/acmi.0.000856.v3

**Published:** 2024-09-04

**Authors:** Constança Bertrand, Rodrigo Martins, Francisco Nunes, Pedro Brandão, Francisco X. Nascimento

**Affiliations:** 1 iBET, Instituto de Biologia Experimental e Tecnológica, Apartado 12, 2781-901 Oeiras, Portugal; 2 ITQB NOVA, Instituto de Tecnologia Química e Biológica António Xavier, Universidade Nova de Lisboa, Av. da República, 2780-157 Oeiras, Portugal

**Keywords:** algae, indole-3-acetic acid, marine, *Marinomonas*

## Abstract

Auxins, mainly in the form of indole-3-acetic acid (IAA), regulate several aspects of plant and algal growth and development. Consequently, plant and algae-associated bacteria developed the ability to modulate IAA levels, including IAA catabolism. In this work, we present and analyse the genome sequence of the IAA-degrading and marine algae-associated bacterium, *Marinomonas* sp. NFXS50, analyse its IAA catabolism gene cluster and study the prevalence of IAA catabolism genes in other *Marinomonas* genomes. Our findings revealed the presence of homologs of the *Pseudomonas iac* gene cluster, implicated in IAA catabolism, in the genome of strain NFXS50; however, differences were observed in the content and organization of the *Marinomonas iac* gene cluster when compared to that of the model *iac*-containing *Pseudomonas putida* 1290. These variations suggest potential adaptations in the IAA catabolism pathway, possibly influenced by substrate availability and evolutionary factors. The prevalence of *iac* genes across several *Marinomonas* species underscores the significance of IAA catabolism in marine environments, potentially influencing plant/algae-bacteria interactions. This study provides novel insights into the IAA catabolism in *Marinomonas*, laying the groundwork for future investigations into the role of *iac* genes in *Marinomonas* physiology and the regulation of marine plant/algae-bacteria interactions.

## Data Summary

The *Marinomonas* sp. NFXS50 (CT5) genome assembly accessed in the National Center for Biotechnology Information(NCBI) database under the GCF_018336975.1 accession number (https://www.ncbi.nlm.nih.gov/datasets/genome/GCF_018336975.1/)

The authors confirm all supporting data, code and protocols have been provided within the article or through supplementary data files.

## Introduction

Phytohormones such as auxins, mainly in the form of indole-3-acetic acid (IAA), are known regulators of plant and algae growth and development. In higher plants, IAA is responsible for the regulation of a wide range of physiological and developmental processes, including root and shoot development, the symbiotic nodulation process and plant biotic and abiotic defence responses [1,2]. In algae (both macro and microalgae), IAA influences a range of physiological and developmental processes, including algal growth regulation, morphogenesis, reproduction and stress responses [3,4]. As a result of the vital role of IAA in shaping plant and algae development, their associated bacterial communities have evolved intricate ways to manipulate IAA levels. In this sense, several plant/algae-associated bacteria are known to synthesize and exude IAA, influencing several aspects of plant/algae-microbe interactions [5,8]. Moreover, recent works have also demonstrated the presence and impact of plant-associated bacteria presenting IAA catabolism activities in shaping plant development and plant-microbe interactions [9,11]. While the beneficial effects of IAA-producing bacteria in plant/algae growth are well documented, not much is understood regarding the prevalence or effects of IAA-degrading bacteria in plant and algal growth and development.

Previous studies have identified the genetic mechanisms involved in the bacterial ability to catabolize IAA and use it as the sole carbon source, which is mainly accomplished by the expression of the *iac* (IAA catabolism) gene cluster [12]. The *iac* cluster was first described in the plant-associated bacterium, *Pseudomonas putida* 1290 [13], and encodes enzymes responsible for the aerobic conversion of IAA to catechol. Functional *iac* clusters were also identified in other plant/soil-associated bacteria such as *Paraburkholderia phytofirmans* PsJN, *Enterobacter soli* LF7 and *Caballeronia glathei* DSM50014 [11,13,16]. Despite the similarity between the *iac* genes found in these bacteria, the *iac* gene cluster organization and composition may vary depending on the bacterial strain [11,16]. Moreover, functional studies have shown the relevance of *iac* genes in the bacterial modulation of plant IAA levels, and its effects on plant development [11,13].

Interestingly, a functional *iac* gene cluster was also found in the marine bacterium, *Marinomonas* sp. MWYL1 [13]. Members of the *Marinomonas* genus (Gammaproteobacteria, Oceanospirilaceae) are usually found in marine environments in association with marine plants and algae [17,18]. The presence of *iac* genes in *Marinomonas* suggests an important role for IAA degradation in the marine environment and in the regulation of the interactions occurring between plants/algae and its associated bacteria. Thus, in an effort to study the role of IAA degradation in marine environments, we have isolated several marine micro-organisms capable of using IAA as the sole carbon source, including *Marinomonas* sp. NFXS50 (CT5). This bacterial strain was found associated with the marine algae, *Codium tomentosum*, obtained from Portuguese coastal seawater, and was capable of using IAA as the sole carbon source (Nascimento *et al*. unpublished results). In this work, we present and analyse the genome sequence of *Marinomonas* sp. NFXS50 and describe its *iac* gene cluster homolog involved in IAA catabolism. Moreover, we demonstrate and discuss the prevalence of the IAA catabolism gene cluster in other members of the *Marinomonas* genus and its potential role in the modulation of algae auxin levels and algae growth.

## Methods

### Genome sequencing and analysis

The genome sequencing of strain NFXS50 was conducted following genomic DNA extraction from an overnight culture grown in Tryptic Soy Broth supplemented with 3% NaCl at 26 °C, using the GenElute bacterial genomic DNA kit (Sigma-Aldrich) according to the manufacturer’s instructions. The genomic library was constructed using the Illumina TruSeq DNA Nano kit and was sequenced using the Illumina’s MiSeq platform and the Illumina’s MiSeq v.3 reagent kit (2×300 bp), generating a total of 937 890 reads. The initial *de novo* genome assembly was performed using SOAPdenovo2 r240 [19]. The final 4 761  082 bp genome sequence of strain NFXS50 was constructed based on a guided assembly against the genome sequence of *Marinomonas* sp. MWYL1 (GenBank accession no. CP000749.1) using MAUVE v.2.4.0 progressive alignments [20]. The contigs were joined by introducing runs of 100 Ns in the identified assembly gap regions, as indicated in the NCBI submission guidelines (https://www.ncbi.nlm.nih.gov/genbank/wgs_gapped/). The genome annotation was performed using the NCBI Prokaryotic Genome Annotation Pipeline v.4.3 (6). The functional annotation of strain NFXS50 genome was conducted using BlastKOALA [21]. Secondary metabolite production clusters were predicted using antiSMASH v.7.1 [22]. Genome maps and analysis were conducted using Proksee [23] and its associated applications, including FastANI [24].

### Comparative analysis of *Marinomonas* sp. NFXS50 IAA catabolism genes

The IAA catabolism genes of *Marinomonas* sp. NFXS50 were identified based on standard blast analysis using the *P. putida* 1290 (GenBank accession no: EU360594) and *Marinomonas* sp. MWYL1 *iac* clusters [13] as query. The *Marinomonas* sp. NFXS50 *iac* gene cluster was then extracted and analysed using the Geneious software [25]. Comparative analysis of *iac* genes and Iac protein sequences was conducted using blast (standard parameters). Multiple alignments between *Marinomonas* sp. NFXS50 and *Marinomonas* sp. MWYL1 *iac* clusters were performed using muscle [26].

### Analysis of the prevalence of *iac* genes in *Marinomonas* genomes

To understand the prevalence of *iac* gene clusters in the *Marinomonas* genus, the available genomes of *Marinomonas* strains were downloaded from the NCBI database (https://www.ncbi.nlm.nih.gov/datasets/genome/?taxon=28253) and analysed. A total of 57 unique Refseq-annotated *Marinomonas* genomes and proteomes (downloaded in April 2024) were obtained and used as reference in standard BLASTp searchers, using the *Marinomonas* sp. MWYL1 Iac protein sequences as the query.

A phylogenetic analysis based on *Marinomonas rpoB* (DNA-directed RNA polymerase subunit beta) genes was constructed to ascertain the phylogenies of the different *Marinomonas* strains and compare the prevalence of *iac* genes in different *Marinomonas* species. The *rpoB* gene was extracted and aligned using muscle v.3.5 [26]. The phylogenetic analysis based on *rpoB* was conducted in mega X [27], using the maximum likelihood method, the GTR+G+I model and a bootstrap analysis of 100 replicates.

## Results

### *Marinomonas* sp. NFXS50 genome main features

**Fig. 1.**
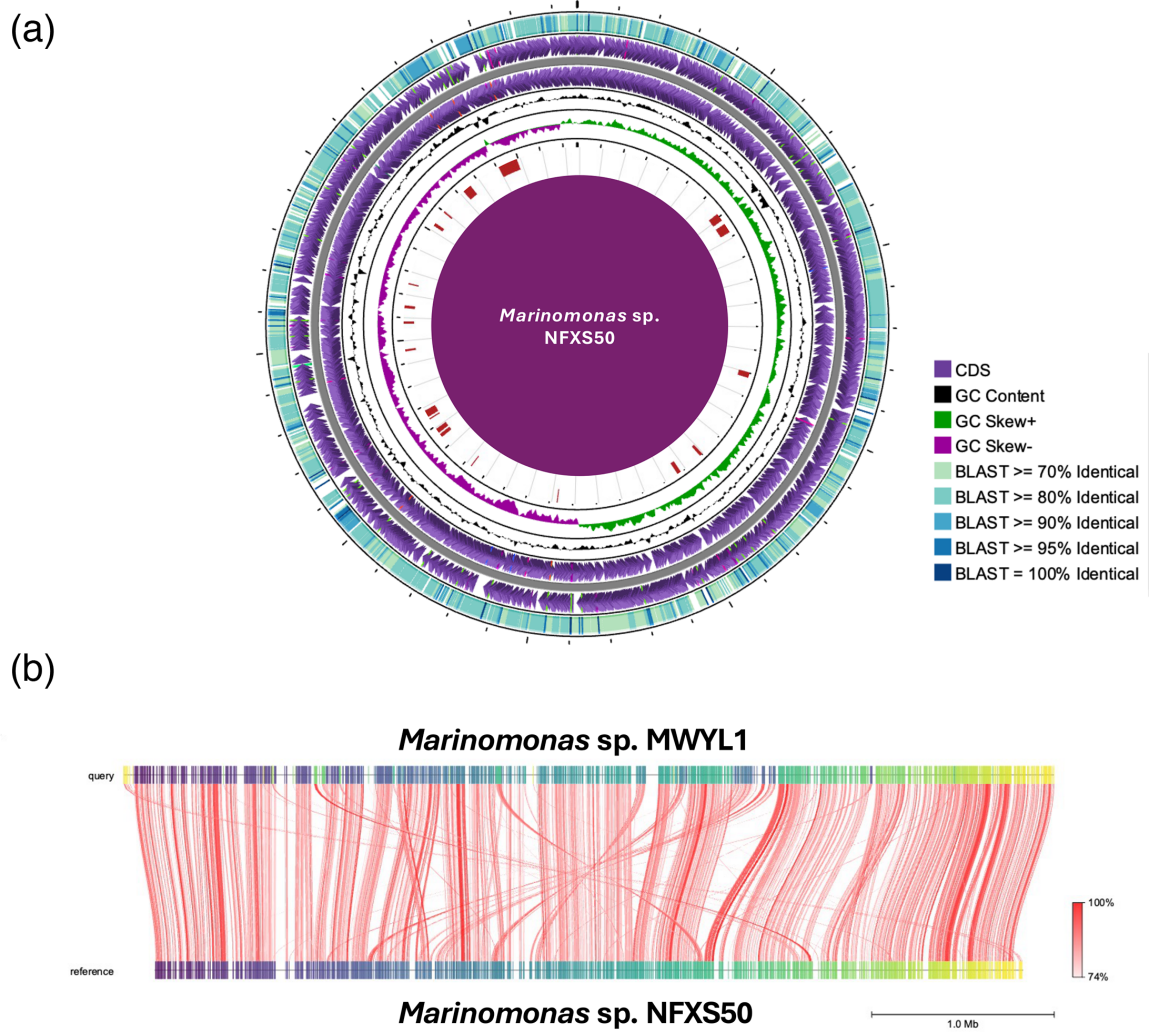
(a) Schematic representation of the *Marinomonas* sp. NFXS50 chromosome, and its similarity to the chromosome of *Marinomonas* sp. MWYL1 (blast analysis). (**b**) Alignment and identity of *Marinomonas* sp. MWYL1 (CP000749.1) and *Marinomonas* sp. NFXS50 (CP025572.1) genomes obtained through FastANI analysis.

The genome of *Marinomonas* sp. NFXS50 is composed by a single circular chromosome of 4.76 Mbp in length with an average GC content of 42.7% (Fig. 1a). A total of 4523 open reading frames were predicted, in which 4387 corresponded to protein-coding sequences (CDSs). A total of 90 RNA-related genes were also detected.

BlastKOALA analysis resulted in the functional annotation of 2525 CDSs (58.1%) in which environmental (350) and genetic (312) information processing functions were assigned for most of the annotated CDSs, followed by carbohydrate (234) and amino acid (206) metabolism. The sec, tat, type I and type II secretion systems were detected in the chromosome of strain NFXS50. The genes encoding the assimilatory sulphate and nitrate reduction pathways were also present in the genome. The analysis conducted in antiSMASH revealed the presence of gene clusters involved in the production of ectoine, one siderophore and two bacteriocins.

Comparative genomic analysis showed that the genomes of *Marinomonas* sp. NFXS50 and *Marinomonas* sp. MWYL1 were highly syntenic (Fig. 1a, b) and presented anAverage Nucleotide Identity (ANI) value of 84.57%. Moreover, BLASTn analysis showed that 4184 from 4387 *Marinomonas* sp. NFXS50 genes were also detected in the genome of *Marinomonas* sp. MWYL1 (Fig. 1a).

### Analysis of *Marinomonas* sp. NFXS50 indole-3-acetic catabolism cluster

**Fig. 2.**
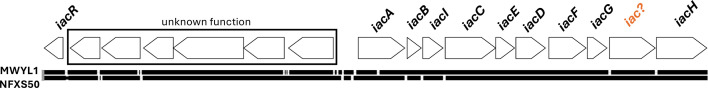
Representation of *Marinomonas* sp. MWYL1 and *Marinomonas* sp. NFXS50 *iac* gene clusters and their overall alignment identity (85.7%).

Homologs of the *iac* gene cluster were detected in the genome sequence of *Marinomonas* sp. NFXS50 (Table 1, Fig. 2). The strain NFXS50 *iac* gene cluster was highly similar (85.7%) to that of the described IAA-degrading bacterium, *Marinomonas* sp. MWYL1 (Fig. 2).

**Table 1.** Comparative analysis of the *Marinomonas* sp. NFXS50 *iac* gene cluster. The identity values correspond to the analysis based on the protein sequences

Locus tag	Product	Gene name	*Marinomonas* sp. MWYL1	*Pseudomonas putida* 1290
**C0J08_14215**	MarR family transcriptional regulator	*iacR*	MMWYL1_RS09225, 95.0%	ABY62764.1, 59%
**C0J08_14210**	Class II aldolase	–	MMWYL1_RS09230, 96.4%	n.f.
**C0J08_14205**	2-Dehydropantoate 2-reductase	–	MMWYL1_RS09235, 89.7%	n.f
**C0J08_14200**	ABC transporter ATP-binding protein	–	MMWYL1_RS092540, 96.0%	n.f
**C0J08_14195**	Metal-dependent hydrolase	–	MMWYL1_RS09245, 95.5%	n.f
**C0J08_14190**	Branched-chain amino acid ABC transporter permease	–	MMWYL1_RS09250, 95.1%	n.f
**C0J08_14185**	Branched-chain amino acid ABC transporter substrate-binding protein	–	MMWYL1_RS09255, 94.0%	n.f
**C0J08_14180**	Acyl-CoA dehydrogenase	*iacA*	MMWYL1_RS09260, 80.9%	ABY62757.1, 62.6%
**C0J08_14175**	IacB protein	*iacB*	MMWYL1_RS09265, 98.3%	ABY62758.1, 78.3 %
**C0J08_14170**	Polyketide cyclase	*iacI*	MMWYL1_RS09270, 93.6%	ABY62766.1, 62.2%
**C0J08_14165**	Rieske (2Fe-2S) protein	*iacC*	MMWYL1_RS09275, 96.5%	ABY62759.1, 65.7%
**C0J08_14160**	Hypothetical protein	*iacD*	MMWYL1_RS09280, 93.1%	ABY62760.1, 48.1%
**C0J08_14155**	3-Oxoacyl-ACP reductase	*iacE*	MMWYL1_RS09285, 98.4%	ABY62761.1, 67.3%
**C0J08_14150**	Oxidoreductase	*iacF*	MMWYL1_RS09290, 95.9%	ABY62762.1, 61.8%
**C0J08_14145**	Flavin reductase	*iacG*	MMWYL1_RS09295, 98.8%	ABY62763.1, 62.8%
**C0J08_14140**	Alpha-hydroxy-acid oxidizing enzyme	–	MMWYL1_RS09300, 96.1%	n.f.
**C0J08_14135**	Amidase	*iacH*	MMWYL1_RS09305, 92.5%	ABY62765.1, 28.6%

n.f.not found

Moreover, both clusters presented a similar organization composed by *iacR*, followed by a cluster containing an aldolase, reductase and amino acid transport genes of unknown function, and finally, the *iacABICDEFG*, an unknown gene and a *iacH* gene homolog. This gene organization was different from that observed in the *P. putida* 1290 *iac* gene cluster (*iacABCDEFGRHI*), as previously described [13]. Despite the different organization, most of the NFX50 *iac* genes and its corresponding protein sequences presented high homology to the *P. putida* 1290 Iac proteins (Table 1). One exception was the *Marinomonas* sp. NFXS50 IacH homolog, which only presented 28.6% identity to the IacH protein of *P. putida* 1290. Despite belonging to the same family (amidase), these proteins presented different sizes (*Marinomonas* sp. NFXS50, 429 aa vs *P. putida* 1290, 374 aa) and overall characteristics.

Moreover, the NFXS50 gene (C0J08_14140, *iac?*) found in the upstream region of the *iacH* homolog was not detected in the * P. putida* 1290 *iac* gene cluster. This gene encoded an alpha-hydroxy acid dehydrogenase family enzyme, presenting similarity with bacterial l-lactate dehydrogenase.

Genomic analysis also revealed the presence of the catechol-1-dioxygenase (C0J08_07140), muconolactone delta-isomerase (C0J08_07135), muconate cycloisomerase (C0J08_07130), 3-oxoadipate CoA-transferase (C0J08_07105–10) and beta-ketoadipyl-CoA thiolase (C0J08_07100) genes in the chromosome of *Marinomonas* sp. NFXS50, consistent with the IAA degradation pathway via catechol.

### Prevalence of IAA catabolism genes in *Marinomonas* genomes

**Fig. 3.**
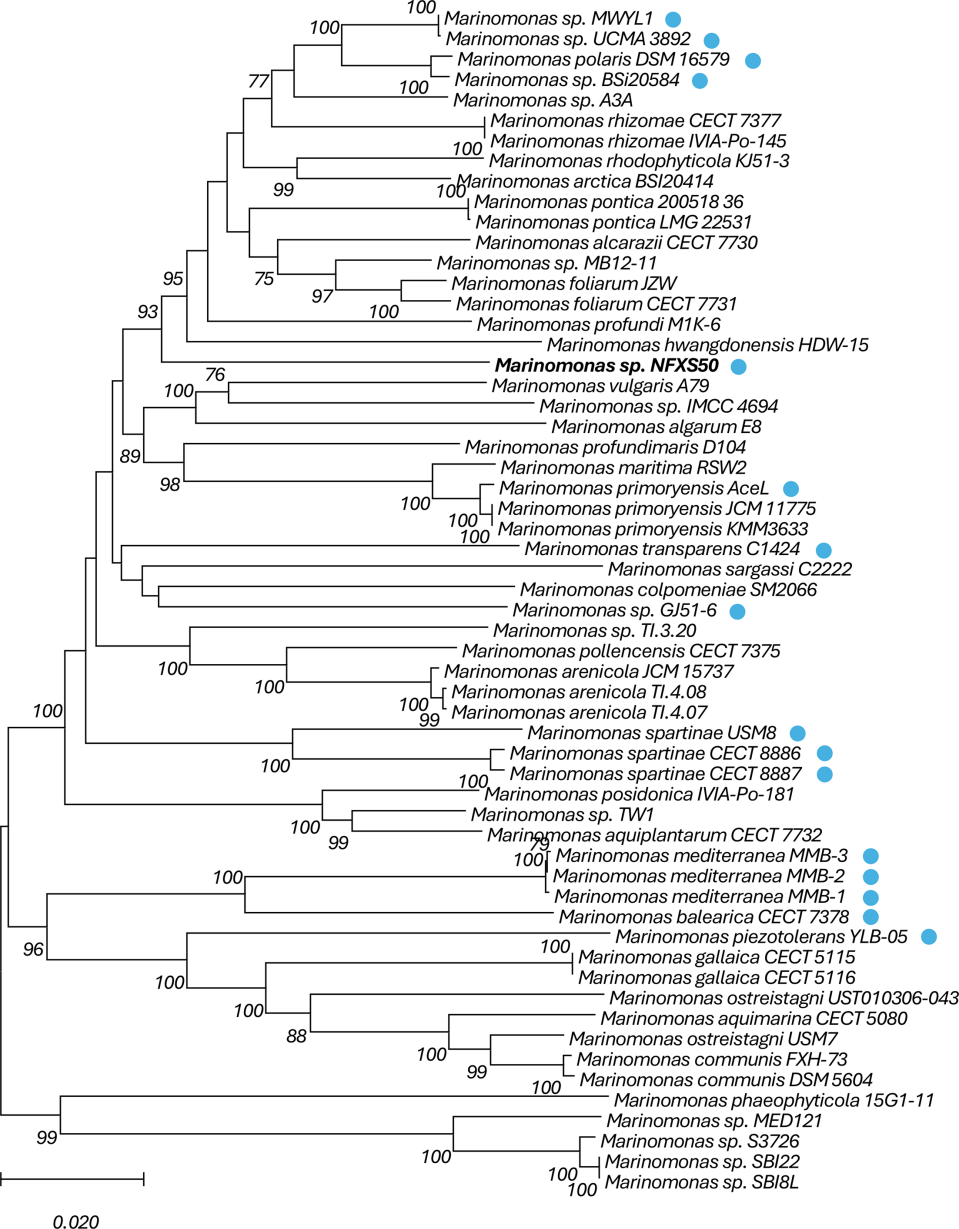
Phylogram based on the *Marinomonas rpoB* gene (housekeeping). Blue circles in front of strain names indicate the presence of the *iac* gene cluster. The tree was constructed using the maximum likelihood method, the GTR+G+I model and a bootstrap analysis of 100 replicates. The tree is rooted at the midpoint.

blast analysis revealed the presence of *iac* gene homologs in 15 of the 57 *Marinomonas* genomes studied (Fig. 3).

The *iac* gene-containing *Marinomonas* belonged to different species (Fig. 3), including *M. spartinae*, *M. mediterranea*, *M. balearica*, *M. piezotolerans*, *M. transparens* and *M. polaris.* The *iac* gene clusters identified in the different *Marinomonas* strains shared an overall pairwise identity of 71.2%.

Interestingly, the *Marinomonas iac* gene alignments showed that some strains present different *iac* gene cluster organization. Overall, the *Marinomonas iac* gene cluster is mostly similar to that described in strains MWYL1 and NFXS50 (Fig. 2); however, some strains lack the gene encoding the alpha-hydroxy acid dehydrogenase family enzyme (*iac?*) in the vicinity of the *iacH* homolog. This was the case of *M. mediterranea* (MMB-1, GCF_000192865.1; MMB-2, GCF_028472805.1; MMB-3, GCF_028472785.1), *M. balearica* (CECT7378, GCF_004362145.1), *M. piezotolerans* (YLB-05, GCF_003362755.1), *M. transparens* (C1424, GCF_016463975.1) and *M. spartinae* (CECT8886, GCF_900089775.1; CECT8887, GCF_900089745.1; USM8, GCF_016461715.1) strains.

## Discussion

In this work, we presented and analysed the genome sequence of the IAA-degrading *Marinomonas* sp. NFXS50. The analysis revealed that strain NFXS50 harbours homologs of the *iac* gene cluster involved in the catabolism of IAA. This result is consistent with previous findings which described the presence of a functional *iac* gene cluster in *Marinomonas* sp. MWYL1 [13]. Comparative analysis showed that *Marinomonas* sp. NFXS50 and *Marinomonas* sp. MWYL1 *iac* gene clusters presented a similar organization and overall identity (85.7%). Nonetheless, the analysis also revealed that the functional *Marinomonas iac* gene cluster presents a different content and organization from that of other *iac*-containing organisms such as *P. putida* 1290 (*iacABCDEFGRHI*) [13]. In this sense, the *Marinomonas iacR* gene, encoding a MarR type transcriptional regulator, was found next to a gene cluster of unknown function that preceded the *iacABICDEFG* cluster. In addition, a gene encoding an alpha-hydroxy acid dehydrogenase family enzyme (*iac?*) and a homolog of the *iacH* gene were detected downstream of the other *iac* genes. Previous works have described the different organization of *iac* genes in distinct bacterial strains [13,16], suggesting different IAA catabolism pathway adaptations related to substrate availability and possibly phylogenetic and evolutionary aspects (e.g. horizontal gene transfer, recombination and genome rearrangements).

Our results also showed that *iac* genes are prevalent in several *Marinomonas* species, with the *iac* gene cluster appearing to be somewhat conserved between the different strains. An exception to this observation was linked to the absence of alpha-hydroxy acid dehydrogenase family enzyme encoding gene (*iac?*) in some of the *Marinomonas* species. The presence of this gene in the *iac* cluster was clearly linked to the observed *Marinomonas* phylogeny, suggesting that evolutionary aspects shaped the *iac* gene cluster content of these strains. Nonetheless, the alpha-hydroxy acid dehydrogenase family enzyme encoding gene was also not found in the *Pseudomonas iac* gene cluster, further suggesting that it is not necessary for the functional IAA catabolism activities.

Curiously, the *Marinomonas iacH* gene homolog presented a very low identity (28.6%) to that of *Pseudomonas*. While encoding an amidase family enzyme, not much is understood about the role of *iacH* in IAA catabolism. Still, it is possible that *iacH* functions in the release of ammonia from an intermediate of the IAA catabolism pathway [12], allowing the producing strain to use IAA as a nitrogen source. In this way, the unusual *Marinomonas iacH and iac* gene organization may reflect its adaptations to IAA catabolism in marine environments. Still, more studies are necessary to unveil the specific function of the *Marinomonas iac* genes in IAA catabolism.

Previous studies have shown that *iac* genes played a role in the capabilities of *P. putida* 1290 and *P. phytofirmans* PsJN to interact with plants [11,13]. The prevalence of *iac* genes in *Marinomonas* indicates that IAA catabolism is also of relevance in the marine environment. *Marinomonas* strains carrying *iac* genes were isolated from seagrass (i.e. *M. spartinae*, *M. balearica*, MWYL1) and algae (i.e. *M. transparens*, NFXS50, GJ51-6), further suggesting that IAA catabolism may impact plant/algae-bacteria interactions in marine environments. This is consistent with the important role of IAA in plant and algae development [1,4]. The *Marinomonas* ability to catabolize IAA suggests that these strains are key players in the positive modulation of marine plant and algae growth and development.

Ultimately, the results obtained in this work bring new insights into the IAA catabolism in *Marinomonas* and pave the way for novel mechanistic studies about the role of the *iac* genes in *Marinomonas* physiology as well in the regulation of marine plant/algae-bacteria interactions.

## Abbreviations

CDSs: coding sequences
IAA: indole-3-acetic acid
NCBI: National Center for Biotechnology Information
